# Protein-specific prediction of mRNA binding using RNA sequences, binding motifs and predicted secondary structures

**DOI:** 10.1186/1471-2105-15-123

**Published:** 2014-04-29

**Authors:** Carmen M Livi, Enrico Blanzieri

**Affiliations:** 1 Department of Information Engineering and Computer Science, University of Trento, Via Sommarive 5, Trento, Italy; 2 Current address: Gene Function and Evolution, Centre for Genomic Regulation (CRG), Dr. Aiguader 88, Barcelona, Spain; 3 Universitat Pompeu Fabra (UPF), Barcelona, Spain

**Keywords:** RNA-protein interaction, Support vector machine

## Abstract

**Background:**

RNA-binding proteins interact with specific RNA molecules to regulate important cellular processes. It is therefore necessary to identify the RNA interaction partners in order to understand the precise functions of such proteins. Protein-RNA interactions are typically characterized using *in vivo* and *in vitro* experiments but these may not detect all binding partners. Therefore, computational methods that capture the protein-dependent nature of such binding interactions could help to predict potential binding partners *in silico*.

**Results:**

We have developed three methods to predict whether an RNA can interact with a particular RNA-binding protein using support vector machines and different features based on the sequence (the *Oli* method), the motif score (the *OliMo* method) and the secondary structure (the *OliMoSS* method). We applied these approaches to different experimentally-derived datasets and compared the predictions with *RNAcontext* and *RPISeq*. *Oli* outperformed *OliMoSS* and *RPISeq*, confirming our protein-specific predictions and suggesting that tetranucleotide frequencies are appropriate discriminative features. *Oli* and *RNAcontext* were the most competitive methods in terms of the area under curve. A precision-recall curve analysis achieved higher precision values for *Oli*. On a second experimental dataset including real negative binding information, *Oli* outperformed *RNAcontext* with a precision of 0.73 *vs.* 0.59.

**Conclusions:**

Our experiments showed that features based on primary sequence information are sufficiently discriminating to predict specific RNA-protein interactions. Sequence motifs and secondary structure information were not necessary to improve these predictions. Finally we confirmed that protein-specific experimental data concerning RNA-protein interactions are valuable sources of information that can be used for the efficient training of models for *in silico* predictions. The scripts are available upon request to the corresponding author.

## Background

The human genome encodes a large number of RNA-binding proteins (RBPs) [1-3] which carry out diverse functions and a range of biological processes. Some RBPs are well characterized, and their molecular functions and biological activities are partially known. RBPs are also involved in post-transcriptional regulation, RNA splicing, RNA stability and protein synthesis. This suggests that RBPs must interact with specific mRNA targets. Each mRNA comprises a coding region flanked by 5’ and 3’ untranslated regions (UTRs). A number of specific RBP target sites have been identified in the 3’-UTR [4]. Interactions between RBPs and RNA are finely tuned and regulated, and the disruption of such interactions is therefore implicated in a number of diseases [3,5]. Furthermore, the identification of RNA targets is interesting from a biological perspective because they provide insight into the precise functions of RBPs [2,6]. More accurate predictions of binding sites and the molecular characteristics of such interactions are therefore highly informative [7].

Over the last decade, several computational approaches have been developed to predict RBP-RNA interactions. One such approach relies on the use of machine learning techniques to predict individual amino acid residues on the protein surface that potentially interact with ribonucleotides, e.g. Neural Networks [8], Random Forest (RF) [9], Naïve Bayes [10] and Support Vector Machines (SVM) [11-13]. These methods are based on information about binding extracted from three-dimensional binding complexes and provide accurate predictions, but they do not consider the RNA-binding partner and provide no information about the RNA sequence that interacts with the RBP. Only a few methods address this issue: (1) Pancaldi and Bähler [14], by predicting RBP-RNA interactions in yeast with SVM and RF; features are global RBP-RNA relationships such as Gene Ontology terms, protein localization information and mRNA properties; (2) *catRAPID*[15], by predicting interactions with long non-coding RNAs on a large-scale using physico-chemical properties and predicted secondary structures; (3) *RPISeq*[16], by predicting whether a given RNA sequence is bound by a specific RBP using pure sequence-derived features combined with the SVM and RF approach; and (4) Wang et al. [17], also using sequence-based features (i.e. protein-RNA interaction propensities) to predict interactions between RBPs and non-coding RNAs on a large-scale by applying Naïve Bayes and Extended Naïve Bayes classifiers. A more detailed description of these methods can be found in a recently published review [18]. Other computational approaches include motif-finding tools that search for binding sites on RNA molecules [1]. These methods need experimental data to extract significant sequence motifs within the bound sequences [19] or to search for relevant sequences and structural motifs by learning from data about bound and non-bound sequences [20].

The interaction between RNA and RBPs is protein specific [21], but the interaction mechanism is not always well described despite many experimental investigations [5,7,22]. This may be due to different binding preferences: some RBPs bind specific target sequences on the RNA strand [2], whereas others recognize their binding site within the RNA secondary structure [23,24]. The binding recognition mechanism may differ even within the same RBP family [22,25]. Currently, the detection of RNA targets and the identification of specific binding sites is only possible by carrying out *in vitro* and *in vivo* experiments such as systematic evolution of ligands by exponential enrichment (SELEX) [26] and the crosslinking and immunoprecipitation (CLIP) techniques [27-29]. These methods are costly, time-consuming, are based on assumptions and have limitations reflecting experimental bias [27,30]. Furthermore the RBP-RNA interactions identified by such techniques tend to be species-dependent or restricted to a particular cell type or set of experimental conditions, and only expressed sequences are detected, which means many non-expressed but genuine interaction partners may be overlooked. The same applies to the non-binding sequence data: the transcriptome in a particular sample does not include all the possible transcripts even in the same species, resulting in the identification of only a subset of the potential binding and non-binding sequences. Computational methods can help to capture specific protein-dependent interactions. Large genome-wide transcription datasets, generated by high-throughput screening, contain valuable information about validated RNA-protein interactions and this information is a useful way to improve *in silico* predictions.

RNA-protein interactions can be predicted by using motif-finding tools to detect RBP-binding sites in RNA sequences, but such methods often fail to detect complex binding mechanisms [30]. A single RNA molecule can contain binding sites for more than one protein [31] and the binding of an RBP can depend on the binding of another protein. Some RBPs may need more than one binding site spread along the folded RNA sequence [30]. These specific binding mechanisms cannot be predicted by motif-finding tools alone but might be caught by features describing the general sequence composition.

We have developed an *in silico* binding prediction tool based on the SVM approach that exploits experimental human datasets. Because each RBP interacts with specific target RNAs [1], it is reasonable to train one SVM per RBP in order to model its specific binding properties. Starting from experimental datasets, we represented each RNA sequence initially by its tetranucleotide composition, followed by the inclusion of significant binding patterns and secondary structure information as features. The use of SVMs was motivated by the superior classifications achieved in previous studies [12,13]. To evaluate the methods, we carried out 10-fold cross validations and compared our results with *RNAcontext*[20] and *RPISeq*[16].

Our novel RBP-dependent approach involves the individual training of one model for each RBP and the exploitation of experimental datasets. The models are trained only on RNA features but also include secondary structure information.

## Methods

### Approach

Our approach comprises the method *Oli* (based on tetranucleotides as features) and two extensions: *OliMo*, which adds protein-specific binding motifs, and *OliMoSS*, which also adds secondary structure information. We applied the proposed methods to experimental human datasets downloaded from The Atlas of UTR Regulatory Activity (AURA) [32]. The well-studied human RBP Pumilio-2 (PUM2), extracted from the Gene Expression Omnibus (GEO) [33], was used to evaluate the influence of true negative RNA sequences on the prediction capability of the models. Additionally, a PAR-CLIP dataset for the RBP Argonaute 2 (AGO2) [27] was downloaded from GEO so that our approaches could be tested independently.

Some RBPs can bind specific sub-sequences (called motifs) but these patterns are not always available. Tools have therefore been developed which search for significant patterns in a group of RNA sequences that are known to interact with the corresponding RBP. In our approach we used MEME Suite [19] to detect binding motifs *in silico* and to embed the binding site information in the form of motif scores.

Other RBPs do not bind sequence motifs but instead recognize secondary structures, which we also included as features. The three-dimensional structure of the protein and the accessibility of the binding site influence the RNA binding [6]. The accessible surface area can be determined by inspecting three-dimensional structures, but there is no high-throughput approach to parse such three-dimensional information. Therefore we introduced a simple accessibility feature: an RNA subsequence was defined as accessible if at least four consecutive nucleotides were single stranded, i.e. not paired with other nucleotides in a stem. Double stranded ribonucleotides would be less accessible to an RBP.

The entire experimental dataset (with bound and non-bound RNA sequences) is available for PUM2, but this does not apply to the remaining RBPs present in *AURA*. Therefore we used 3000 human 3’-UTRs, randomly downloaded from the Ensembl Genome Browser [34], as negatives. It is possible that these negatives include transcripts that were not detected in the experiment but are potentially bound by the RBP, e.g. because they were not bound under the given conditions or their expression level was low. This fact makes the choice of negative training data challenging and it can also influence the results. Therefore we calculated the confidence interval of the obtained areas under the curves (AUCs) by exchanging the 3000 human 3’-UTRs 10 times with other randomly-selected transcripts from ENSEMBL.

The application of machine learning to biological data is often affected by unbalanced datasets, because the number of negative examples is generally much higher than the positive ones. We chose 3000 3’-UTRs as negatives, because this is double the greatest number of sequences used in the *AURA_dataset*, and small enough to train models in a reasonable time. Several solutions have been proposed to address the issue of unbalanced datasets [35] and we decided to use an oversampling algorithm called SMOTE [36], which creates new synthetic instances within the positive data and forces the classifier to become more general.

To study our methods we carried out two different evaluations. In Evaluation Evaluation 1 we assessed the prediction of *Oli*, *OliMo* and *OliMoSS* against 15 different RBPs using randomly-selected 3’-UTRs as negatives. In Evaluation Evaluation 2, to assess the influence of experimentally-validated and artificial negative data, we applied our methods to a protein with available experimentally-determined non-bound sequences. The comparison of empirical negative data and randomly-selected 3’-UTRs for PUM2 allowed us to determine the value of using real negative training data.

In both evaluations we compared our approaches to *RPISeq*[16] and *RNAcontext*[20]. *RPISeq* is directly comparable to our methods because it uses protein and RNA sequences as inputs and predicts binding using SVM (*RPISeq-SVM*) and Random Forest (RF) (*RPISeq-RF*). Similarly, *RPISeq* applies the normalized tetranucleotide frequency to describe the RNA sequences.

*RNAcontext* uses a different approach based on the detection of sequence motifs and structures in a pool of training sequences, and searches for them in a set of test sequences before assigning a score.

### Datasets

All the datasets were derived from experiments with human cells.

#### 

##### 

**AURA_dataset.** The *AURA_dataset* comprises RBPs and related RNA sequences downloaded from AURA (release 2.4). AURA is an online database that contains experimentally-derived human mRNA-RBP pairs. For simplicity, ’*RBP+*’ refers to the set of RNA sequences in AURA that are recognized by a specific RBP. For example *CPEB1+* comprises 182 RNA sequences recognized by CPEB1 and *PUM1+* comprises 420 RNA sequences recognized by PUM1. An *AURA_dataset* with 15 *RBP+* sets was obtained by focusing on proteins with more than 50 associated 3’-UTRs (enough positive examples to train SVM) for which MEME Suite was able to detect binding motifs in a reasonable time. In order to eliminate similar sequences we processed each *RBP+* with USEARCH [37] to cluster sequences with more than 80% (and 30%) identity. We used one representative from each cluster as the final sequence. By randomly choosing 3000 human 3’-UTRs from the Ensembl Genome Browser [34] we constructed an artificial negative dataset (hereafter called *3K-*) to complement each *RBP+* set. Thus for CPEB1 the *CPEB1+* set comprised 182 (positive) binding sequences and *3K-* was used as the negative data to train the SVM.

##### 

**PUM2_dataset.** The PUM2 data originates from a photoactivatable ribonucleoside-enhanced CLIP experiment on human embryonic kidney (HEK293) cells and was downloaded from GEO (GSM545210). In the experiment [28], 7523 clusters of about 3000 transcripts were identified and 93% were found within the 3’-UTRs. We extracted all 3’-UTRs in such way that each cluster appeared only once, creating the *PUM2+* dataset which contains 2151 positive 3’-UTRs recognized by PUM2. The results of an RNA-Seq experiment [27] involving the same HEK293 cells under the same conditions were used as the negative results. Two replicates are available on GEO: GSM714678 and GSM714679. To avoid the loss of data we merged these results and downloaded the sequences from the Ensembl Genome Browser (NCBI36/hg18 release 54, May 2009). Hence the *PUM2-* dataset comprises 3000 of the 12329 negative 3’-UTRs that did not bind to PUM2.

##### 

**iAGO2_dataset.** The independent AGO2 (iAGO2) dataset is not present in AURA and was downloaded from GEO as replicates of the PAR-CLIP experiment [27]: GSM714644 and GSM714645. Merging the results and downloading the sequences from the Ensembl Genome Browser (NCBI36/hg18 release 54, May 2009) resolved 5951 sequences recognized by AGO2. The RNA-Seq dataset discussed above was again used to provide the negative 3’-UTRs [27]. After subtracting the transcripts recognized by AGO2, the negative dataset comprised 5841 sequences.

Table 1 gives a short description of the datasets composition, the number of proteins and the number of target sequences. A more detailed description of all RBPs can be found in Additional file 1. The data for the *AURA_dataset*, *3K-*, *PUM2+*, *PUM2-* and *iAGO2* are provided in Additional file 2.

**Table 1:** Dataset description

**Dataset**		**No. proteins**	**No. of targets**	**Type**
*AURA_dataset*				
	RBP+	15	8086	positive dataset
	*3K-*	-	3000	negative dataset
*PUM2_dataset*				
	*PUM2+*	1	2151	positive dataset
	*PUM2-*	1	3000	negative dataset
*iAGO2_dataset*				
		1	5951	positive dataset
		1	5841	negative dataset

A short description of the dataset compositions, the number of proteins in each dataset and the sum of the target sequences. A more detailed description of the *AURA_dataset* and the number of target sequences used for training can be found in Table 2, first and second column.

**Table 2:** **Performance of** ***Oli*,** ***OliMo*,** ***OliMoSS*,** ***RNAcontext* and** ***RPISeq* on the** ***AURA_dataset***

**Name**	**#(*RBP+*)**	***Oli***	***OliMo***	***OliMoSS***	***RNAcontext***	***RPISeq-SVM***	***RPISeq-RF***
AGO1	1824	0.86	0.85	0.84	0.83	0.74	0.62
AGO2	207	0.84	0.83	0.70	0.80	0.7	0.61
AGO4	270	0.87	0.84	0.78	0.82	0.76	0.62
AUF1	1319	0.69	0.69	0.67	0.62	0.57	0.6
CPEB1	182	0.69	0.67	0.59	0.55	0.62	0.53
CPEB4	72	0.52	0.54	0.60	0.50	0.54	0.52
CUGBP1	195	0.78	0.78	0.65	0.72	0.72	0.6
ELAVL1	1262	0.73	0.73	0.69	0.68	0.6	0.61
PUM1	420	0.68	0.68	0.66	0.68	0.67	0.64
PABP	258	0.57	0.58	0.52	0.52	0.52	0.51
QKI	710	0.87	0.86	0.86	0.83	0.78	0.76
TNRC6A	246	0.87	0.83	0.79	0.82	0.67	0.67
TNRC6B	742	0.86	0.86	0.82	0.83	0.70	0.68
TNRC6C	151	0.80	0.80	0.68	0.77	0.70	0.61
U2AF65	228	0.73	0.73	0.67	0.71	0.64	0.64
Mean ±sd		0.75 ±0.11	0.75 ±0.10	0.70 ±0.09	0.71 ±0.11	0.66 ±0.07	0.61 ±0.06

The table lists RBPs, the number of sequences and the AUCs for each method on the *AURA_dataset*. The AUCs are calculated in 10-fold cross validations and at a sequence identity of 80%. The negatives are provided in all cases by *3K-* (see Evaluation Evaluation 1). Data are reported with means ± standard deviation (sd).

### SVM

The method we use to classify binding and non-binding RNA sequences is the Support Vector Machine (SVM) [38]. An SVM classifier tries to discriminate linearly between RNA sequences which belong to different classes *y*_*i*_ with *y*_*i*_∈{+1,−1}: a sequence *x*_*i*_ belongs to the positive class with label +1 (e.g. bound RNA) or to the negative class with label −1 (e.g. not bound RNA). The goal of a SVM is to find a discrimination function, which divides the two classes in such way that the label for new entries can be predicted. In this work, we use the freely available SVM package LIBSVM [39].

### Feature extraction and representation

#### PSSM

Motifs are sequence patterns in RNA, DNA or proteins that can be modeled by position-specific scoring matrices (PSSMs). MEME Suite [19] can detect motifs in sets of sequences, create the corresponding PSSM and compute the motif score ${\text{score}}_{{ŝ}_{i}}$ which is calculated for each *m*-length subsequence ${ŝ}_{i}=b_{i+1}\ldots b_{i+m}$, *i*∈{0,...,*n*−*m*+1} along the n-length RNA sequence *b*_1_*b*_2_…*b*_*j*_…*b*_*n*_ where *b*_*j*_ is the ribonucleotide at the *j*-th position and *m* the motif length *m*≤*n*:

(1) $${\text{score}}_{{ŝ}_{i}}=\overset{m}{\underset{k=1}{\sum }}\text{pssm}(b_{i+k},k)$$

where *pssm*(*b*,*k*) returns the matrix value for *b*∈{*A*,*U*,*C*,*G*} and position *k*.

We searched for significant motifs in each *RBP+* set using the following MEME Suite property settings: mod = zoops, minw = 5 and maxw = 10.

#### Tetranucleotides

We codified the individual RNA sequence compositions using the frequency of all possible tetranucleotides (*AAAA*,*AAAU*,*AAUC*,…). The corresponding feature reported the frequency of each tetranucleotide in the overall RNA sequence.

#### Simple secondary structures

We evaluated the following features based on RNA secondary structures predicted with RNAfold [40]: 

1. predicted folding energy [14] (calculated using RNAfold);

2. stem density, proportion of paired base pairs [14];

3. number of stems [14];

4. accessibility, computed by identifying subsequences with at least four consecutive nucleotides in single stranded form, i.e. not part of a stem. We codified these subsequences using tetranucleotides. The corresponding feature was set to 1 if a specific subsequence was single stranded, but otherwise 0. Additional file 3: Figure S1 illustrates the calculation of the secondary structure features.

### Prediction methods

Using *Oli*, binding and non-binding RNAs for a specific RBP were predicted by applying a SVM that described each RNA sequence in terms of the frequency of 256 features corresponding to all possible tetranucleotide sequences.

*OliMo* extended *Oli* by adding 10 PSSM-based motif scores. Given a specific RBP, we applied a SVM to discriminate binding from non-binding RNAs, describing each RNA sequence using 266 features: the tetranucleotide frequencies (see above) and 10 PSSM-based motif scores calculated for each subsequence in the RNA strand. Binding sites occur more often than expected by chance on regulated UTRs [4]. Accordingly we represented the binding sites on each RNA sequence by the 10 highest motif scores.

*OliMoSS* extended *OliMo* by adding secondary structure features. Given a specific RBP, we applied a SVM to discriminate binding from non-binding RNAs by describing each RNA sequence with a total of 525 features: the tetranucleotide frequencies and PSSM scores described above, plus three additional secondary structure properties (the predicted folding energy of the formed secondary structure, the stem density and the number of stems in the structure) and 256 features representing the accessibility of different tetranucleotides.

### Evaluation and comparison

The models were analysed in two evaluations. In **Evaluation **Evaluation 1, we tested the predictive capability of *Oli*, *OliMo* and *OliMoSS* against the *AURA_dataset*, each *RBP+* set assisted with the negative *3K-* counterpart, and calculated the AUC and precision, as previously reported with *RNAcontext* and *RPISeq*. Finally we applied the Wilcoxon signed-rank test (with a significance level of 0.01) to the AUCs to compare their performance. In order to assess the significance of the resulting AUCs, we calculated their confidence interval: we carried out the experiment 10 times, each time generating the *3K-* set using 3000 randomly selected and non-overlapping ENSEMBL transcripts. Furthermore, we investigated whether the trained protein-specific SVMs discriminate between the different *RBP+* sets. To address this, we applied each model to the *RBP+* sets of the other 14 RBPs and calculated the sensitivity. We also used *iAGO2* to test the predictions of our AGO2-models. **Evaluation **Evaluation 2 tested the performance of *Oli*, *OliMo* and *OliMoSS* on *PUM2+* compared to *PUM2-* and *3K-*. We calculated AUC and precision, and compared the predictions (using the Wilcoxon signed-rank test) with *RNAcontext* and *RPISeq*.

A short description and a definition of the performance metrics can be found in the Additional file 3.

In each evaluation we carried out a 10-fold cross validation. Within each training fold, a grid-search identified the best value for the linear kernel parameter *C* according to the highest Matthews correlation coefficient (MCC) to evaluate the classification ability. An n-fold cross-validation is preferable to a leave-one-out cross-validation because it has been used in most previous reports [9,12,13,16,17] and is recommended for calculations that are demanding on computer resources [41]. Moreover, Muppirala et al. and Pancaldi and Bähler reported no differences in the predictions compared to the use of leave-one-out cross-validation. All the scripts we describe were implemented in Python. The oversampling algorithm SMOTE was used to balance the data and was applied only to the training folds. We sought binding motifs with MEME Suite in each of the 10 training folds separately to avoid circularity and to ensure a fair testing. Here, we only applied the linear kernel because the balancing with SMOTE was implemented in the input space and the linear kernel must therefore be used. Other kernels would require the training data to be balanced in each kernel-defined feature-space, and this transformation was not the goal of the paper.

## Results and discussion

### Evaluation 1

Table 2 shows the performance of *Oli*, *OliMo*, *OliMoSS*, *RNAcontext*, *RPISeq-SVM* and *RPISeq-RF* on each RBP in the *AURA_dataset*. Each *RBP+* set was filtered using sequence identity thresholds of 80% (results shown in Table 2) and 30% (results shown in Additional file 3: Table S2). High levels of sequence identity in the 10-fold cross validation can introduce biases and can shift the results versus high true positive and true negative predictions. This is generally the case for protein sequences but evidently does not apply to RNA sequences. Lower levels of sequence identity did not influence our predictions, thus we only report the results for the *RBP+* sets with less than 80% sequence identity. It was not possible to generate *RPISeq* predictions for the 30% identity dataset because the method is restricted to 100 RNA sequences per run and is only accessible online. It is not feasible to calculate interactions within large datasets in steps of 100 RNA sequences. For this reason we calculated the AUCs only for the sequences with 80% identity.

Additional file 3: Table S1 reports the confidence intervals of the resulting AUCs at a confidence level of *α*=0.01. The p-values of the Wilcoxon signed-rank test are presented in Additional file 3: Table S3, and the precisions for each method, calculated at a threshold of 0.5, can be found in Additional file 3: Table S4.

*Oli* and *OliMo* achieved the highest mean AUC of 0.75, followed by *RNAcontext* with a mean of 0.71. *RPISeq-SVM* and *RPISeq-RF* performed the worst, with means of 0.66 and 0.61, respectively. The Wilcoxon signed-rank test showed a significant difference between the prediction of *Oli* and *OliMoSS* (p = 0.004) and between the prediction of *OliMo* and *OliMoSS* (p = 0.006) but there was no significant difference between *Oli* and *OliMo* (p = 0.202). All three approaches showed a statistically significant difference in prediction compared to *RPISeq-RF*. *RNAcontext* showed statistically significant differences in prediction to *RPISeq-SVM* (p = 0.01) and *RPISeq-RF*(p = 0.001). Similarly, *Oli* and *OliMo* are statistically different from *RNAcontext* (p = 0.001 in each case) and from both *RPISeq* methods (p < 0.002). All approaches were characterized by low precision values, the mean ranging from 0.14 (*RPISeq-RF*) to 0.34 (*Oli* and *OliMo*). *RNAcontext* (Prec = 0.29) and *OliMoSS* (Prec = 0.30) outperformed both *RPISeq-SVM* (Prec = 0.15) and *RPISeq-RF* (Prec = 0.14). The computation of the precision at a threshold of 0.5 does not show the overall potential of the methods, which is instead best visualized by PR curves.

*Oli* was compared in detail with *RNAcontext*, because the latter was the most competitive with our novel methods. To visualize the classification ability of the two approaches, we plotted the PR curve (Additional file 4) and the receiver operating characteristic (ROC) curve (Additional file 5) for each RBP. The optimum area of a PR curve is the upper-right corner, and both methods struggled to reach it for proteins CPEB4 and PABP. *Oli* outperformed *RNAcontext* for most RBPs and the curve was visibly shifted over the y-axis. Regarding the ROC curves, both approaches were competitive, essentially reflecting the AUCs listed in Table 2.

The performance of our approaches was protein dependent. For several RBPs (e.g. AGO1, AGO2, AGO4 and QKI), *Oli* and *OliMo* achieved an *AUC*≥0.80, whereas in other cases (e.g. CPEB4 and PABP), the performance was worse (*AUC*≤0.6). This is possibly because each RBP binds in a specific way and the adopted features may not always capture the particular binding property.

For example, Argonaute family members are known to bind small miRNAs which finally bind the target sequences. This complementarity is probably easier to detect in the binding sequences, which could explain the good performances of AGO1, AGO2 and AGO4. Similarly, protein quaking (QKI) binds to RNA targets containing the core sequence -YUAAY- [42]. When applied to the poly(A)-binding protein (PABP), which predominantly binds to the poly-A tails of mRNAs, our methods generated nearly random results. One explanatory hypothesis is that the poly-A sequence alone is not enough to discriminate between positive and negative data.

**Figure 1:**
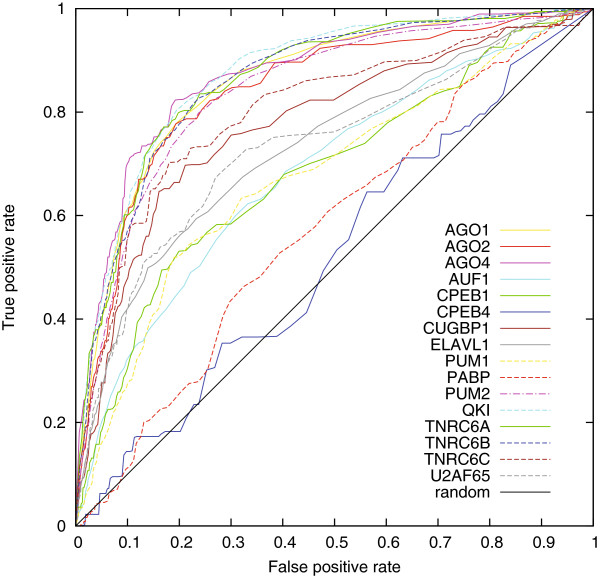
**ROC curve showing the performance of tetranucleotide frequency-based discrimination.** The ROC curves show the performance in 10-fold cross validations for the *Oli* method on the *AURA_dataset* and on *PUM2+*. The negative data are in both cases provided by *3K-*. The further the ROC curve advances towards the upper-left corner, the better the classification ability of the model. A curve near the 45-degree diagonal reflects a random classification.

We anticipated that as more binding information is provided, such as motif score and secondary structure, better discrimination would be achieved between binding and non-binding RNA. However, we observed the opposite phenomenon in our models. *OliMoSS* achieved only low AUCs and a statistically significant difference in prediction, confirming it was the weakest of our approaches. We concluded that the secondary structure features were not necessary, perhaps because enough binding information is already included in the tetranucleotide representation. Furthermore, the accessibility feature can also have a limited impact because some RBPs bind RNA backbones rather than accessible ribonucleotides. Generally the tetranucleotide-based features were able to capture the specific binding properties. Figure 1 shows the performance of *Oli* on the *AURA_dataset*.

We also tested the widely-used normalization of tetranucleotide features, as applied in *RPISeq*, but this did not improve either the AUC or the precision (data not shown). Normalizing the tetranucleotide features can disrupt the frequency of important tetranucleotides and thus reduce discrimination within our dataset. Further improvements can instead be achieved by applying other rebalancing techniques. For example, the undersampling of *3K-* in combination with the oversampling algorithm SMOTE for the positive data, proved to be a better choice for smaller datasets with fewer than 50 sequences (the cut-off value we used). An alternative balancing technique is the assignment of different weights to different classes during training (implemented in *LIBSVM*). However, weighting the classes did not influence the predictions achieved using our data.

Here we used the tetranucleotide frequencies to code the RNA sequences. These features are identical to *RPISeq*. Previously published studies use dinucleotides [43] to identify contact profiles from RBP-RNA complexes and Wang et al. [17] chose sequences, three nucleotides in length, to codify RNA. In our case, using k-mers of length two and four gave similar prediction results, whereas using k-mers larger than six reduced the prediction ability of the classifiers and caused much higher calculation times for the balancing algorithm *SMOTE*. Using longer k-mers to represent the RNA sequences forces most of the frequencies to be 0. A similar result was observed in our dataset for RNAs shorter than 1500 nucleotides: sensitivity and specificity are unbalanced because most of the tetranucleotide frequencies are zero. Interestingly, highly unbalanced datasets, with many more negative sequences than positives, did not have such an impact. The calculated AUC remained stable even if the negative dataset size increased substantially. This suggests that even in an unbalanced scenario, the proportion of correctly predicted sequences remains balanced.

Although applying the same sequence features, our protein-specific discrimination (i.e. one model for each RBP) was more useful that the general discrimination approach of *RPISeq*. Because proteins utilise different binding mechanisms, a protein-specific model can catch binding preferences better than a less specific one. Specific models can be created by detecting and isolating important and protein-dependent features. To gain more insight, we calculated the information gain [44] for each tetranucleotide. The 18 most important features for each protein, ranked by their information gain, can be found in the Additional file 3: Table S5. An interesting observation is that the highest values are assigned to UUUU, AAAA, AUUU and UUUA (in contrast to Muppirala et al., who reported AUUC, AGUG, UUUU and UCAA as the most frequent tetranucleotides) and that the 10 highest-ranked tetranucleotides do not really vary across the proteins. The protein-dependent tetranucleotides only begin to differ in the lower ranks. We argue that these results can be used as the first criteria to select important features and to create more specific models.

The binding mechanism is not the only factor that differs between proteins and needs individual treatment. The datasets themselves also differ, because they reflect different experiments with various cell-lines under diverse conditions. One single model created using such a mix of information and diversity, cannot reasonably be expected to achieve accuracy, whereas protein-dependent training can catch the precious information contained in each dataset.

To investigate whether the protein-specific SVMs discriminate between the different *RBP+* sets, we calculated the sensitivity and specificity for each RBP model. Assuming each RNA in the *RBP+* sets as a positive (i.e. binding-partner), we can calculate the sensitivity of the model against the binding partners of the other RBPs. The results are shown in Additional file 3: Table S7. Interestingly, many protein models detected binding sequences (with sens >0.5) in the QKI, AGO1-4 and TNRC6A-C datasets. AUF1 detected binding transcripts in all *RBP+* sets. The opposite phenomenon was observed for CPEB4 and PABP: the sensitivities never exceed 0.4. Transcripts have binding sites for several RBPs, therefore it is possible to find targets also within the other *RBP+* sets. Moreover, by assuming that the shared and overlapping targets are positives and the non-shared targets are negatives, we calculated the sensitivities and specificities for all the models. The results can be found in Additional file 3: Table S8 for the sensitivities and Additional file 3: Table S9 for the specificities. As expected, the sensitivities in this test are high, because the binding partners have been detected also in the other *RBP+* sets, and almost all of the models achieved low specificities on *RBP+* sets AGO1-4 and TNRC6A-C. The number of overlaps between the bound sequences in the different *RBP+* sets can be found in Additional file 3: Table S6.

Inferring RBP-RNA binding based only on the presence of specific binding motifs may underestimate the complexity of the binding process for some RBPs, explaining the lower performance of *RNAcontext* in the PR curves. The same applies in the case of *OliMo*, which did not improve the predictions even when motif-based features were included. Motif-based features may work better when known and experimentally verified motifs can be used. Considering that high-throughput methods produce large amounts of data, even a small change in the precision of an *in silico* method results in the better prediction of binding RNAs.

The *iAGO2_dataset* was used to test our AGO2-models independently: *Oli* performed best (AUC = 0.71), followed by *OliMo* (AUC = 0.69) and *OliMoSS* (AUC = 0.63). These values are promising and show that models trained on experimental data can be useful to discriminate between target sequences. The availability of further high-throughput data will make it possible to test more of our models independently.

### Evaluation 2

Table 3 compares the performance of *Oli*, *OliMo*, *OliMoSS*, *RNAcontext*, *RPISeq-SVM* and *RPISeq-RF* on *PUM2+* and two different negative datasets: *PUM2-* and *3K-*. The ROC curve for *PUM2+* and *3K-* is shown in Figure 1. *Oli* and *OliMo* achieved similar AUCs and precision on the *PUM2-* and *3K-* datasets, and both performed marginally better than *OliMoSS*. *RNAcontext* and *RPISeq-SVM* achieved similar AUCs to *Oli* and *OliMo* on both datasets, but much lower precision scores. The worst performance was achieved by *RPISeq-RF* (AUC <0.57 and Prec <0.43). Secondary structure features did not improve the prediction, confirming the results of Evaluation Evaluation 1. We expected models trained on real binding data (i.e. *PUM2-*) to increase the degree of discrimination, but instead this reduced the performance of all methods.

**Table 3:** **Performance of** ***Oli*,** ***OliMo*,** ***OliMoSS*,** ***RNAcontext* and both** ***RPISeq* methods on** ***PUM2+* in combination with two different negative datasets**

**Pos. data**	**Neg.data**	**Value**	***Oli***	***OliMo***	***OliMoSS***	***RNAcontext***	***RPISeq-SVM***	***RPISeq-RF***
*PUM2+*	*PUM2-*	AUC	0.77	0.77	0.74	0.75	0.73	0.52
		Prec	0.73	0.73	0.69	0.59	0.48	0.42
*PUM2+*	*3K-*	AUC	0.84	0.84	0.82	0.83	0.77	0.56
		Prec	0.80	0.80	0.74	0.68	0.47	0.40

The table shows the performance for each method on two different datasets: one with *PUM2+* and experimental negatives *PUM2-* and one with *PUM2+* and randomly selected 3’-UTRs *3K-*. AUC and precision (Prec) values were calculated for a 10-fold cross validation.

In order to determine the ability of a model based on real negative data to find binding partners among general 3’-UTR sequences, we tested the models generated by the 10-fold cross validation with *PUM2+* and *PUM2-*, substituting the negatives of each of the 10 sets with negatives from *3K-*. Evidently an approach based on real data should also be able to distinguish between real positives and randomly-selected sequences. Furthermore, the task should be easier than distinguishing real negatives because the precision increased (Prec = 0.81). This is consistent with the fact that all the methods perform better when the negatives are sourced from the *3K-* dataset. Similarly, to determine the ability to distinguish between real positives and real negatives, we considered the models trained with *PUM2+* and *3K-* and tested their performance against *PUM2-*. In this case, the precision declined to 0.69. Therefore a complete dataset obtained by *in vivo* experiments can be used effectively to train SVMs with simple sequence features.

The evaluation highlights the importance of negative training data, which is rarely available but is necessary to build accurate models. Because the performance values of all methods tested on *PUM2-* and *3K-* are correlated with a Pearson coefficient of 0.99 (Table 3), random sequences can provide a good approximation if no experimental negatives are available. They can also be used to determine the relative performance of the methods, as shown in Evaluation Evaluation 1.

**Figure 2:**
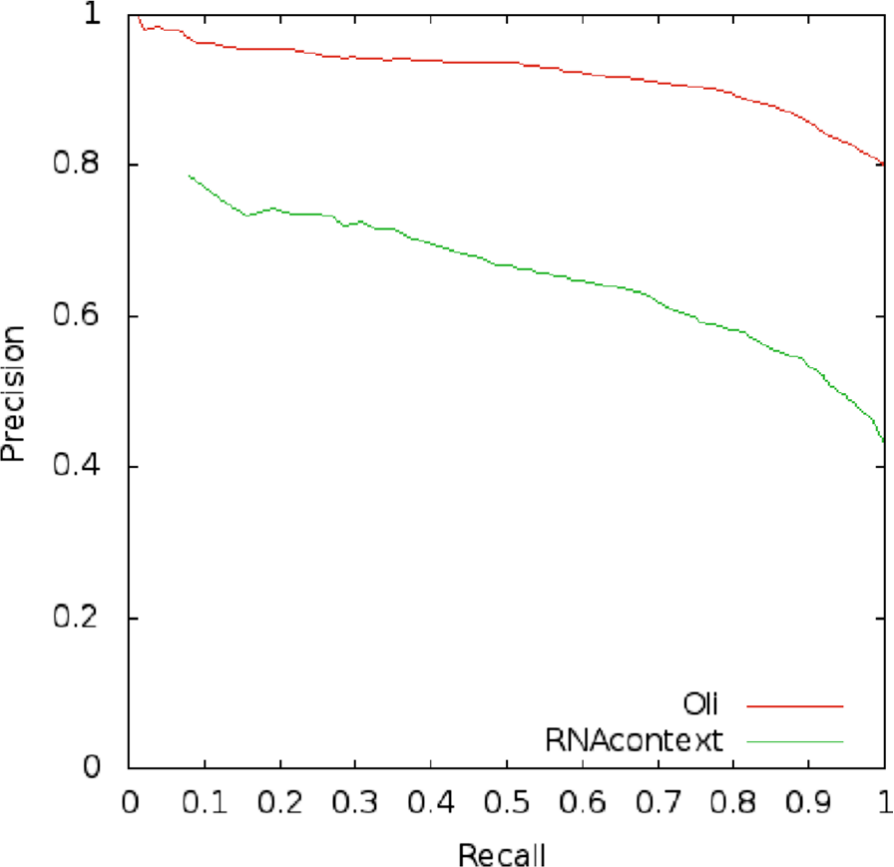
**Precision-recall curves of*Oli* and*RNAcontext*.** The PR curves show the performance of *Oli* method (red line) and *RNAcontext* (green line) on experimental data from *PUM2+* and *PUM2-* using 10-fold cross validations. The further the curve advances to the upper-right corner, the better the classification ability of the model. A more detailed explanation is provided in the Additional file 3.

Finally, when training with *PUM2-*, the difference in precision is 0.14 between *Oli* and *RNAcontext* and 0.25 between *Oli* and *RPISeq-SVM*. As discussed under Evaluation Evaluation 1, even a small change in precision is important. If we consider 1000 RNAs, an increase in precision of 0.14 results in the correct classification of an additional 140 RNA sequences. Figure 2 shows the PR curve for *Oli* and *RNAcontext* on *PUM2+* and *PUM2-*.

## Conclusions

The correct characterization of RBP-RNA interactions *in silico* provides important data for the assignment of protein functions, which currently requires either *in vivo* and *in vitro* laboratory experiments. We applied SVMs to experimental datasets and attempted to predict RNA targets for different RBPs. We initially described the RNA sequences in three different ways: (1) the *Oli* method, which uses tetranucleotides as features; (2) the *OliMo* method, which also incorporates motif scores from automatically-detected binding motifs; and (3) the *OliMoSS* method, which extends *OliMo* by also including secondary structure features. We compared the predictions achieved using our methods with those generated by *RNAcontext* and *RPISeq*. *Oli* and *OliMo* performed better than *OliMoSS* and *RPISeq* and when applying the same nucleotide-based features *Oli* outperformed *RPISeq*, supporting our decision to train SVMs for each RBP separately. Binding motifs alone were not discriminative enough on our datasets, as shown by the higher precision of *Oli* and *OliMo* compared to *RNAcontext*. Further comparisons showed that models trained on randomly-chosen RNA sequences performed better than those trained on experimentally-detected non-binding sequences. Experimental data can therefore be used to train an SVM with tetranucleotide frequency features, which can then be used to predict interactions with other RNA sequences.

An ideal method should predict RBP interactions in the absence of existing binding data. Thus we are aware of a limitation in our proposed work: interactions can only be predicted when at least one experimental dataset for an RBP exists. This goal still remains challenging. Future work may include the incorporation of accessibility features calculated according to nucleotide solvent accessibility [45] and not only, as in our case, by the tetranucleotide single-stranded form, as well as the creation of protein-dependent models trained with pre-selected RBP-specific features.

We conclude that simple sequence information, such as tetranucleotide representation within an RNA sequence, in combination with experimental binding data, can be used effectively to construct accurate predictive models. However, the choice of negative training examples is important. They can be approximated using random sequences if real data are not available, but ideally they should be derived from the same experiment, under the same conditions, and using the same cell line. Only under these conditions would computational methods be able to capture specific binding phenomena to identify the precise discriminative properties of a given protein.

## Competing interests

The authors declare that they have no competing interests.

## Authors’ contributions

CML and EB designed the study. CML implemented the algorithm and conducted the computational experiments. Both authors wrote and revised the manuscript. All authors read and approved the final manuscript.

## Supplementary Material

Additional file 1 **RBP descriptions.** The table contains a description of all RBPs used in the *AURA_dataset* and a description of the PUM2 protein. The file is in csv format and tab-delimited. The first column lists the UniProt ID of the RBP followed by the protein name, gene name and the function (source: UniProt [46]). Click here for file

Additional file 2 **Datasets.** This tab-delimited table is in csv format and contains the RNA sequences for each *RBP+* set, for *3K-*, *PUM2+*, *PUM2-* and *iAGO2*. The first row shows RBP names followed by the bound RNA identifiers (if not otherwise specified, this is an UCSC Genome Browser ID [47]). Click here for file

Additional file 3 **Supplementary Information.** This pdf file contains the description of the performance measures, supplementary figure and tables. Click here for file

Additional file 4 **Precision-recall curves for** ***Oli* and** ***RNAcontext* on the** ***AURA_dataset.*** This pdf file shows a PR curve for each RBP, visualizing the performances of *Oli* and *RNAcontext* in a 10-fold cross validation. *Oli* outperforms *RNAcontext* for most RBPs. Click here for file

Additional file 5 **ROC curves for** ***Oli* and** ***RNAcontext* on the** ***AURA_dataset.*** This pdf file shows a ROC curve for each RBP, visualizing the performances of *Oli* and *RNAcontext* in a 10-fold cross validation. The curves basically reflect the AUCs in Table 1 and do not show a significant difference in the prediction ability of the two methods. Click here for file
