# Supplementary material for: Protein-specific prediction of mRNA binding using RNA sequences, binding motifs and predicted secondary structures
Source: BMC Bioinformatics. 2014 Apr 29;15:123. doi: 10.1186/1471-2105-15-123 (PMC4098778; doi:10.1186/1471-2105-15-123)
Supplement: Additional file 3 — Supplementary Information. This pdf file contains the description of the performance measures, supplementary figure and tables. [file 1471-2105-15-123-S3.pdf]

Supplementary Information

Performance measures

A binary classifier, such as SVM, assigns to predicted binding sequences the positive class label (+1) and to sequences predicted as non-binding the negative class label (−1). Correct assignments to the positive or the negative class increase the numbers of the true positives (TP) or the true negatives (TN), respectively. When wrongly attributed, false negatives (FN) or false positives (FP) increase.

Matthews correlation coefficient (MCC) is a balanced measure and indicates the correlation between observed and predicted classification.

$$MCC = \frac{TP \cdot TN - FP \cdot FN}{\sqrt{(TP + FP) \cdot (TP + FN) \cdot (TN + FP) \cdot (TN + FN)}} \tag{1}$$

The **precision (Prec)**, also called positive predictive value, indicates the portion of positive classified examples that are really positive:

$$Prec = \frac{TP}{TP + FP}. \tag{2}$$

If each element in the data is assigned to the negative class then TP and FP are zero. In this case the precision is not defined any more.

The **sensitivity (Sens)** indicates the fraction of right classified examples in the positive class:

$$Sens = \frac{TP}{TP + FN}. \tag{3}$$

The creation of the **receiver operating characteristic (ROC)** curve is a common way to visualize a model performance. The x-axis shows the false positive rate and the y-axis displays the true positive rate by varying a parameter, in our case the classification threshold. The true positive rate and false positive rate are defined as

$$TPR = \frac{TP}{TP + FN} \tag{4}$$

and

$$FPR = \frac{FP}{FP + TN}, \tag{5}$$

respectively.

A similar visualization gives the **Precision-Recall (PR)** curve showing the precision on the y-axis and the recall on the x-axis:

$$Recall = \frac{TP}{TP + FN}. \tag{6}$$

By calculating the **area under the ROC curve (AUC)** the performance of a classifier can be reduced to a single value. A reasonable classifier has an $AUC \geq 0.5$, a perfect one an AUC of 1.

Figure S1: Calculation of secondary structure features

The secondary structure features are calculated in the following way:
A) RNAfold is used to predict the secondary structure of the transcript. RNAfold also calculates the predicted folding energy, which is directly used as an independent feature. For each secondary structure we calculate the stem density as the ratio of the number of base paired nucleotides and the total RNA length. The feature *number of stems* is simply the count of stems in the sequence. B) The accessibility is computed by identifying subsequences with at least four consecutive nucleotides in single stranded form, which do not form part of a stem. If a tetranucleotide is accessible, the corresponding feature is set to 1. If at least one nucleotide of the tetranucleotide is paired, the corresponding feature is set to 0.

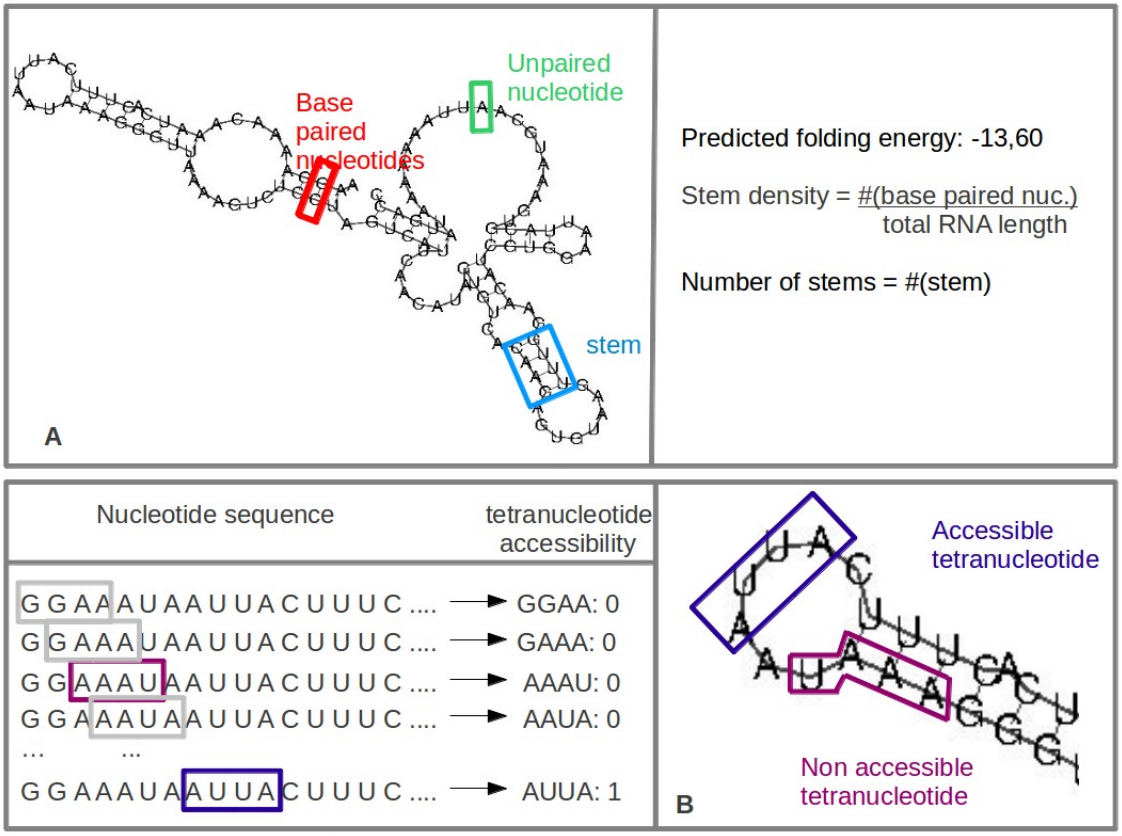

Table S1 - Confidence interval of the AUCs on the *AURA_dataset*

The table lists the RBP name, the AUC and its confidence interval (in square brackets) at a confidence level of $\alpha = 0.01$, for each method on the *AURA_dataset*. The confidence interval is calculated for each AUC by exchanging *3K*- 10 times with other randomly selected non-overlapping transcripts from ENSEMBL.

Name	*Oli*	*OliMo*	*OliMoSS*
AGO1	0.86 [0.94,0.96]	0.85 [0.94,0.95]	0.84 [0.93,0.95]
AGO2	0.84 [0.90,0.94]	0.83 [0.90,0.95]	0.70 [0.80,0.85]
AGO4	0.87 [0.92,0.94]	0.84 [0.92,0.95]	0.78 [0.94,0.96]
AUF1	0.69 [0.68,0.71]	0.69 [0.68,0.71]	0.67 [0.65,0.67]
CPEB1	0.69 [0.70,0.73]	0.67 [0.72,0.77]	0.59 [0.58,0.63]
CPEB4	0.52 [0.31,0.43]	0.54 [0.39,0.51]	0.60 [0.39,0.51]
CUGBP1	0.78 [0.75,0.80]	0.78 [0.73,0.81]	0.65 [0.60,0.62]
ELAVL1	0.73 [0.75,0.77]	0.73 [0.73,0.76]	0.69 [0.66,0.69]
PUM1	0.68 [0.71,0.76]	0.68 [0.70,0.78]	0.66 [0.60,0.63]
PABP	0.57 [0.43,0.49]	0.58 [0.45,0.51]	0.52 [0.43,0.51]
QKI	0.87 [0.97,0.98]	0.86 [0.97,0.98]	0.86 [0.97,0.98]
TNRC6A	0.87 [0.94,0.97]	0.83 [0.93,0.97]	0.79 [0.90,0.95]
TNRC6B	0.86 [0.90,0.92]	0.86 [0.94,0.96]	0.82 [0.89,0.92]
TNRC6C	0.80 [0.85,0.89]	0.80 [0.89,0.92]	0.68 [0.80,0.85]
U2AF65	0.73 [0.82,0.88]	0.73[0.83,0.86]	0.67 [0.80,0.83]

Table S2 - Performance of ***Oli***, ***OliMo***, ***OliMoSS*** and ***RNA-context*** on the ***AURA_dataset*** with a sequence identity of 30%

The table lists RBPs, the number of sequences and the AUCs achieved using each method on the *AURA_dataset*. The AUCs are calculated in 10-fold cross validations and at a sequence identity of 30%. The negatives are provided in all cases by *3K-*. Data are reported with means ± standard deviation (sd).

Name	#(*RBP+*)	*Oli*	*OliMo*	*OliMoSS*	*RNAcontext*
AGO1	1728	0.86	0.86	0.84	0.83
AGO2	177	0.81	0.80	0.67	0.80
AGO4	237	0.85	0.85	0.74	0.82
AUF1	1112	0.67	0.67	0.62	0.62
CPEB1	162	0.69	0.67	0.63	0.55
CPEB4	60	0.42	0.41	0.57	0.50
CUGBP1	173	0.79	0.79	0.69	0.72
ELAVL1	1043	0.71	0.71	0.69	0.68
PUM1	371	0.71	0.73	0.65	0.68
PABP	235	0.53	0.58	0.51	0.52
QKI	602	0.86	0.86	0.83	0.83
TNRC6A	242	0.85	0.86	0.78	0.82
TNRC6B	646	0.83	0.83	0.81	0.83
TNRC6C	136	0.79	0.76	0.65	0.77
U2AF65	200	0.72	0.7	0.63	0.71
Mean±sd		0.73±0.12	0.73±1.12	0.68±0.09	0.71±0.11

Table S3 - Wilcoxon signed-rank test

The table shows the results of the Wilcoxon signed-rank test for each method over all AUCs.

	Oli	*OliMo*	*OliMoSS*	*RNAcontext*	*RPISeq-SVM*	*RPISeq-RF*
Oli	X	0,202	0.004	0.001	0.001	0.001
OliMo		X	0.006	0.001	0.001	0.0007
OliMoSS			X	0.469	0.032	0.0007
RNAcontext				X	0.010	0.001
RPISeq-SVM					X	0.009

Table S4 - Precision values for the *AURA_dataset*

The table contains the precision values calculated in a 10-fold cross validation for each RBP. The last row shows the mean and the standard deviation of the precision values for each method.

Name	*Oli*	*OliMo*	*OliMoSS*	*RNAcontext*	*RPISeq-SVM*	*RPISeq-RF*
AGO1	0.80	0.81	0.74	0.68	0.40	0.38
AGO2	0.28	0.28	0.22	0.23	0.07	0.07
AGO4	0.37	0.38	0.27	0.30	0.09	0.09
AUF1	0.41	0.42	0.41	0.50	0.33	0.32
CPEB1	0.13	0.13	0.09	0.06	0.06	0.06
CPEB4	0.03	0.04	0.06	0.02	0.02	0.02
CUGBP1	0.20	0.20	0.14	0.18	0.07	0.06
ELAVL1	0.61	0.73	0.74	0.55	0.34	0.31
PABP	0.10	0.10	0.08	0.07	0.08	0.08
PUM1	0.29	0.29	0.23	0.19	0.14	0.12
QKI	0.57	0.47	0.49	0.50	0.21	0.21
TNRC6A	0.29	0.32	0.27	0.29	0.08	0.08
TNRC6B	0.55	0.56	0.48	0.50	0.21	0.20
TNRC6C	0.21	0.20	0.17	0.15	0.06	0.05
U2AF65	0.23	0.23	0.18	0.18	0.07	0.07
Mean±sd	0.34±0.21	0.34±0.22	0.30±0.22	0.29±0.20	0.15±0.12	0.14±0.11

Table S5 - Information gain ratio of tetranucleotides in the *AURA_dataset*

The information gain ratio is calculated on the tetranucleotides for each protein in the *AURA_dataset*. The table shows the 18 most important tetranucleotides for each protein, i.e. tetranucleotides ranked by the information gain ratio.

AGO1	AGO2	AGO4	AUF1	CPEB1	CPEB4	CUGBP1	ELAVL1	PABP	PUM1	QKI	TNRC6A	TNRC6B	TNRC6C	U2AF65
UUUU	UUUU	UUUU	UUUU	UUUU	UUUU	UUUU	UUUU	UUUU	UUUU	UUUU	UUUU	UUUU	UUUU	UUUU
AAAA	AAAA	AAAA	AAAA	AAAA	AAAA	AAAA	AAAA	AAAA	AAAA	AAAA	AAAA	AAAA	AAAA	AAAA
AUUU	AUUU	AUUU	AUUU	AUUU	AUUU	AUUU	AUUU	AUUU	AUUU	AUUU	AUUU	AUUU	AUUU	AUUU
UUUA	UUUA	UUUA	UUUG	AAAU	UUCU	UUUA	UUUA	AAAU	UUUA	UUUA	UUUA	UUUA	UUUG	UUUA
AAAU	AAAU	AAAU	CUUU	CUUU	UUUG	UUUG	UUUG	CCUG	AAAU	AAAU	UUUG	AAAU	UAAA	UUUG
UUUG	UUUG	UUUG	UUUA	UAAA	CCUG	AAAU	CUUU	UUUG	UUUG	UAAA	AAAU	UUUG	AAAU	AAAU
UAAA	CUUU	UAAA	UUCU	UUUA	AAAU	CUUU	AAAU	UUUA	UAAA	UUUG	CUUU	UAAA	UUUA	CUUU
UUAA	UAAA	UUAA	UUUC	UUUG	UUUA	UAUU	UUUC	UUCU	CUUU	UUAA	UUAA	CUUU	CUUU	UUUC
CUUU	UUCU	CUUU	AAAU	UUUC	AGAA	UAAA	UUCU	UAAA	UUCU	CUUU	UUCU	UUAA	UUAA	UUCU
UAUU	UUAA	UUUC	UGUU	UUCU	UAAA	UGUU	UGUU	CUUU	UGUU	UAUU	UGUU	UUUC	UUGU	UAAA
UUUC	UGUU	UGUU	CCUG	GAAA	CUGU	UUAA	UAAA	UUUC	UUAA	AAUU	UUUC	UAUU	UUUC	UGUU
UGUU	UUUC	UAUU	GUUU	AGAA	CUUU	UUUC	GUUU	CUGG	UUUC	UUUC	UAAA	UGUU	UAUU	GUUU
UUCU	UAUU	GUUU	UAAA	UGUU	CCAG	GUUU	UUGU	UGUG	UAUU	UUCU	UAUU	UUCU	UUCU	UAUU
AAUU	AAAG	AAUU	UCUU	UAUU	UCUU	UUCU	UAUU	AGAA	UUGU	UGUU	GUUU	GUUU	UGUU	UUAA
GUUU	UUGU	UUCU	UUGU	AAAG	UUUC	UUGU	UCUU	UGUU	AAUU	UUGU	UUGU	AAUU	GUUU	UCUU
UUGU	AAUU	UUAU	UGUG	AAUU	AAAG	AAUU	UUAA	CUGU	GUUU	GUUU	UUAU	UUGU	UUAU	UUGU
UUAU	GAAA	UUGU	CUGG	CUGU	CCCA	UCUU	AAUU	CCAG	UCUU	UUAU	GAAA	UCUU	UCUU	AAUU
GAAA	AGAA	UCUU	CUGU	UUAA	UCUG	UUAU	GAAA	GAAA	UUAU	GAAA	UCUU	UUAU	AAUU	UGUG

Table S6 - Number of shared and overlapping binding partners for all *RBP+* sets in the *AURA_dataset*

The table shows the number of shared target sequences for all *RBP+* sets.

transcripts	Applied model-SVM														
	AGO1 1824	AGO2 207	AGO4 270	AUF1 1319	CPEB1 182	CPEB4 72	CUGBP1 195	ELAVL1 1262	PUM1 420	PABP 258	QKI 710	TNRC6A 246	TNRC6B 742	TNRC6C 151	U2AF65 228
AGO1 1824		134	234	103	11	4	27	134	69	15	288	158	404	88	38
AGO2 207	134		54	10	0	0	3	11	7	2	38	37	61	27	3
AGO4 270	234	54		22	3	0	6	32	16	2	51	65	108	40	5
AUF1 1319	103	10	22		21	9	29	593	68	33	29	21	60	12	32
CPEB1 182	11	0	3	21		21	5	18	4	0	7	0	4	1	4
CPEB4 72	4	0	0	9	21		2	10	0	0	2	0	1	2	2
CUGBP1 195	27	3	6	29	5	2		48	22	10	11	6	10	1	4
ELAVL1 1262	134	11	32	593	18	10	48		72	39	46	27	70	14	26
PUM1 420	69	7	16	68	4	0	22	72		29	24	14	29	7	12
PABP 258	15	2	2	33	0	0	10	39	29		5	0	8	1	4
QKI 710	288	38	51	29	7	2	11	46	24	5		56	129	31	10
TNRC6A 246	158	37	65	21	0	0	6	27	14	0	56		94	33	6
TNRC6B 742	404	61	108	60	4	1	10	70	29	8	129	94		47	17
TNRC6C 151	88	27	40	12	1	2	1	14	7	1	31	33	47		3
U2AF65 228	38	3	5	32	4	2	4	26	12	4	10	6	17	3	

Table S7 - Sensitivity calculated on the overlapping sequences for each *RBP+* set for *AURA_dataset*.

The sensitivities are shown for SVM models trained on *RBP+* sets, shown in the columns, and applied on the *RBP+* sets of the RBPs, shown in the rows. All the sequences in the *RBP+* sets are considered binding partners and the sensitivity of the model is calculated.

		Applied model-SVM														
		AGO1	AGO2	AGO4	AUF1	CPEB1	CPEB4	CUGBP1	ELAVL1	PUM1	PABP	QKI	TNRC6A	TNRC6B	TNRC6C	U2AF65
transcripts	AGO1		0.64	0.61	0.71	0.70	0.19	0.64	0.56	0.77	0.35	0.65	0.69	0.70	0.62	0.58
	AGO2	0.58		0.61	0.69	0.74	0.23	0.61	0.57	0.78	0.29	0.68	0.70	0.71	0.64	0.57
	AGO4	0.67	0.74		0.70	0.75	0.17	0.69	0.65	0.81	0.35	0.70	0.75	0.76	0.70	0.62
	AUF1	0.21	0.25	0.24		0.37	0.19	0.34	0.43	0.38	0.37	0.26	0.29	0.30	0.23	0.28
	CPEB1	0.23	0.31	0.26	0.65		0.28	0.34	0.36	0.41	0.36	0.30	0.31	0.31	0.29	0.32
	CPEB4	0.18	0.25	0.17	0.64	0.36		0.22	0.32	0.31	0.31	0.26	0.24	0.25	0.25	0.28
	CUGBP1	0.44	0.48	0.48	0.76	0.55	0.18		0.54	0.64	0.43	0.50	0.52	0.51	0.45	0.47
	ELAVL1	0.30	0.34	0.33	0.77	0.45	0.20	0.42		0.46	0.38	0.35	0.39	0.39	0.32	0.35
	PUM1	0.33	0.36	0.36	0.66	0.45	0.18	0.44	0.40		0.38	0.37	0.41	0.40	0.35	0.39
	PABP	0.11	0.12	0.12	0.59	0.26	0.16	0.24	0.24	0.26		0.12	0.17	0.16	0.12	0.16
	QKI	0.62	0.66	0.62	0.71	0.75	0.21	0.64	0.57	0.80	0.37		0.72	0.72	0.65	0.61
	TNRC6A	0.62	0.68	0.65	0.71	0.70	0.24	0.62	0.65	0.77	0.33	0.67		0.72	0.65	0.62
	TNRC6B	0.58	0.65	0.61	0.70	0.72	0.18	0.63	0.61	0.76	0.37	0.65	0.69		0.64	0.60
	TNRC6C	0.56	0.62	0.62	0.68	0.66	0.19	0.58	0.56	0.77	0.33	0.61	0.66	0.68		0.56
	U2AF65	0.45	0.48	0.43	0.68	0.57	0.17	0.48	0.44	0.58	0.40	0.50	0.50	0.51	0.50	

Table S8 - Sensitivity calculated only on shared binding partners between each *RBP+* set for *AURA_dataset*

The sensitivities are calculated for SVMs trained on *RBP+* sets shown in the columns and applied only on the overlapping sequences of *RBP+* sets, shown in the rows. The shared sequences in the *RBP+* sets are considered positives and used for calculations.

	Applied model-SVM														
transcripts	AGO1	AGO2	AGO4	AUF1	CPEB1	CPEB4	CUGBP1	ELAVL1	PUM1	PABP	QKI	TNRC6A	TNRC6B	TNRC6C	U2AF65
AGO1		0.82	0.77	0.85	0.81	0.75	1.00	0.87	0.89	0.93	0.87	0.81	0.81	0.78	0.94
AGO2	0.65		0.77	0.90	n.o.	n.o.	1.00	0.10	1.00	0.50	0.92	0.81	0.75	0.7	1
AGO4	0.68	0.81		0.81	1.00	n.o.	1.00	0.90	0.93	1.00	0.86	0.83	0.80	0.75	1
AUF1	0.65	0.90	0.68		0.38	0.88	0.79	0.54	0.51	0.69	0.75	0.66	0.70	0.66	0.62
CPEB1	0.81	n.o.	0.66	0.71		0.95	0.20	0.38	1.00	n.o.	0.71	n.o.	0.75	0	0.5
CPEB4	0.50	n.o.	n.o.	0.66	0.47		0.50	0.30	n.o.	n.o.	1.00	n.o.	1.00	0	0.5
CUGBP1	0.92	1.00	1.00	0.79	0.20	0.50			0.68	0.80	0.90	1.00	1.00	0	0.75
ELAVL1	0.76	0.81	0.87	0.81	0.38	0.70	0.79	0.68	0.63	0.69	0.89	0.85	0.77	0.78	0.73
PUM1	0.69	1.00	0.81	0.83	0.75	n.o.	0.72	0.59		0.62	0.87	0.92	0.82	0.71	0.75
PABP	0.53	0.00	0.00	0.72	n.o.	n.o.	0.70	0.48	0.55		0.80	n.o.	0.62	1	0.25
QKI	0.76	0.92	0.88	0.82	0.71	1.00	1.00	0.86	0.91	0.80		0.89	0.83	0.74	1
TNRC6A	0.72	0.83	0.81	0.90	n.o.	n.o.	1.00	0.88	0.92	n.o.	0.89		0.81	0.69	0.83
TNRC6B	0.69	0.77	0.78	0.93	0.75	1.00	1.00	0.85	0.93	1.00	0.84	0.80		0.72	0.88
TNRC6C	0.68	0.74	0.82	0.91	0.00	0.50	1.00	0.85	1.00	1.00	0.70	0.78	0.72		1
U2AF65	0.86	1.00	1.00	0.84	0.75	1.00	0.75	0.76	0.75	1.00	1.00	0.66	0.82	1	

Table S9 - Specificity calculated on each *RBP+* set for *AURA_dataset*

The specificities are shown for SVMs trained on *RBP+* sets (column) and applied on the non-overlapping sequences contained in the other *RBP+* sets (row). All non-overlapping binding sequences in the *RBP+* sets are considered negatives and the specificities are calculated.

		Applied model-SVM													
		AGO1	AGO2	AGO4	AUF1	CPEB1	CPEB4	CUGBP1	ELAVL1	PUM1	PABP	QKI	TNRC6A	TNRC6B	TNRC6C	U2AF65
transcripts	AGO1		0.38	0.42	0.30	0.30	0.81	0.37	0.46	0.23	0.66	0.39	0.32	0.34	0.39	0.43
	AGO2	0.55		0.44	0.32	0.26	0.77	0.39	0.46	0.23	0.71	0.38	0.33	0.30	0.37	0.43
	AGO4	0.44	0.28		0.31	0.25	0.83	0.31	0.39	0.20	0.66	0.33	0.28	0.27	0.31	0.38
	AUF1	0.82	0.75	0.77		0.63	0.81	0.67	0.66	0.63	0.64	0.75	0.71	0.72	0.77	0.73
	CPEB1	0.81	0.69	0.74	0.35		0.81	0.66	0.64	0.60	0.64	0.72	0.69	0.70	0.71	0.68
	CPEB4	0.84	0.75	0.83	0.37	0.69		0.79	0.68	0.69	0.69	0.76	0.76	0.76	0.74	0.73
	CUGBP1	0.64	0.53	0.53	0.25	0.44	0.82		0.51	0.36	0.59	0.53	0.50	0.52	0.55	0.54
	ELAVL1	0.76	0.66	0.68	0.27	0.55	0.80	0.59		0.55	0.63	0.67	0.62	0.63	0.68	0.66
	PUM1	0.74	0.65	0.66	0.38	0.55	0.82	0.57	0.64		0.64	0.66	0.61	0.63	0.66	0.62
	PABP	0.92	0.88	0.88	0.43	0.74	0.84	0.78	0.81	0.77		0.89	0.83	0.86	0.88	0.84
	QKI	0.49	0.35	0.40	0.30	0.25	0.80	0.37	0.45	0.21	0.63		0.29	0.30	0.35	0.40
	TNRC6A	0.58	0.34	0.41	0.31	0.30	0.76	0.39	0.38	0.24	0.67	0.39		0.34	0.36	0.38
	TNRC6B	0.55	0.36	0.42	0.32	0.28	0.83	0.38	0.41	0.25	0.64	0.39	0.33		0.37	0.41
	TNRC6C	0.60	0.40	0.46	0.34	0.34	0.82	0.42	0.47	0.24	0.67	0.42	0.37	0.35		0.45
	U2AF65	0.64	0.53	0.58	0.35	0.44	0.84	0.52	0.60	0.43	0.61	0.52	0.50	0.52	0.51	
