# Supplementary figures and images for: Protein-specific prediction of mRNA binding using RNA sequences, binding motifs and predicted secondary structures
Source: BMC Bioinformatics. 2014 Apr 29;15:123. doi: 10.1186/1471-2105-15-123 (PMC4098778; doi:10.1186/1471-2105-15-123)

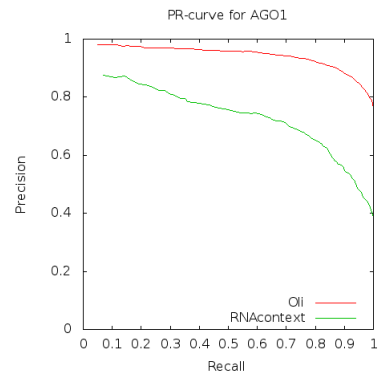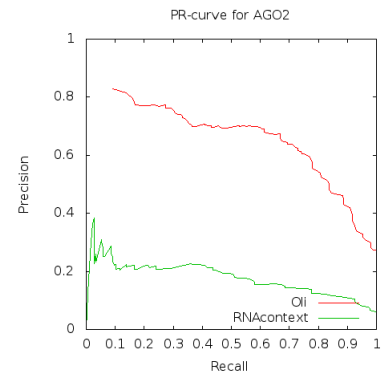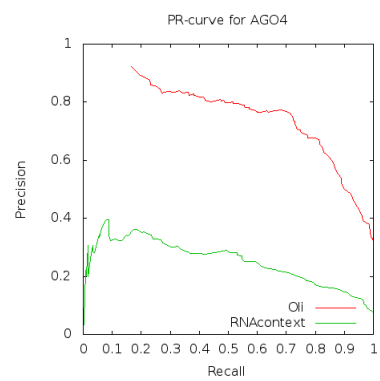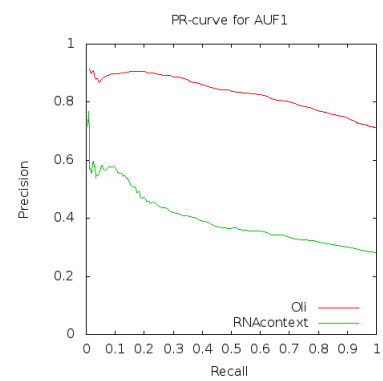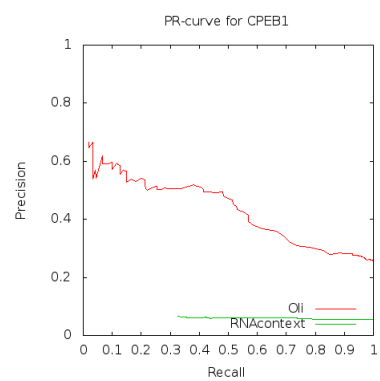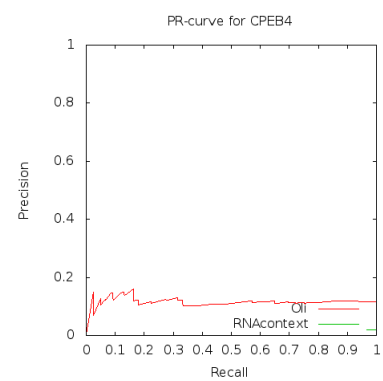

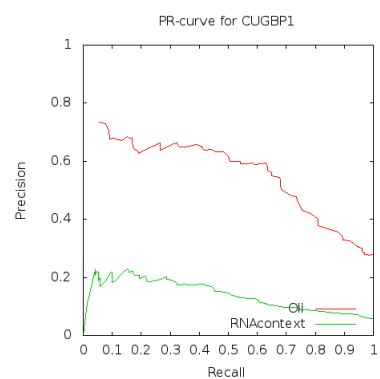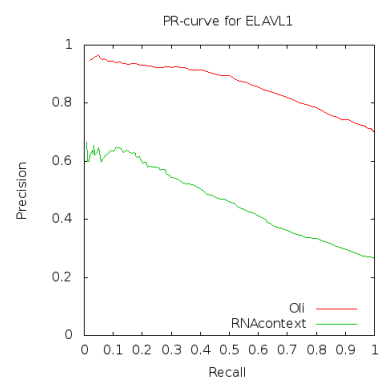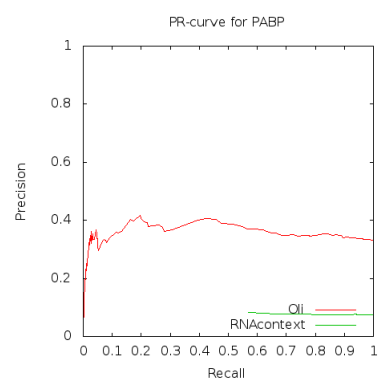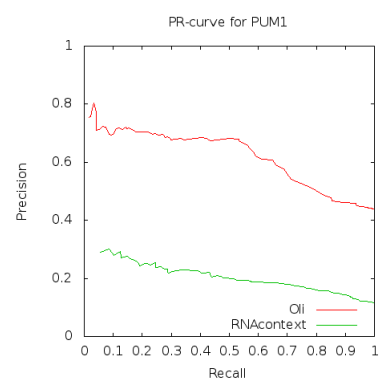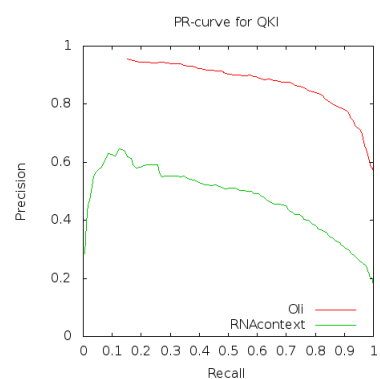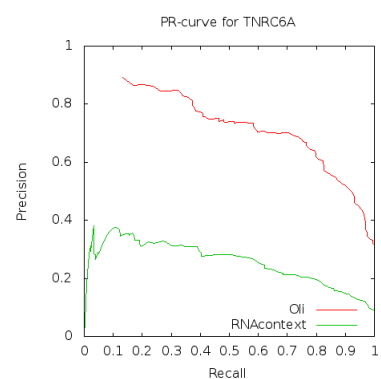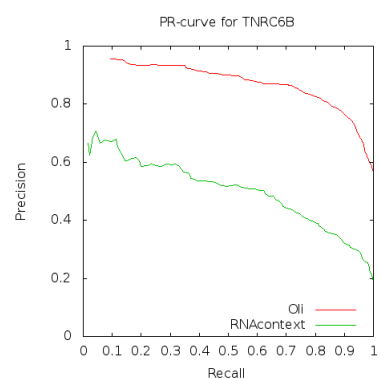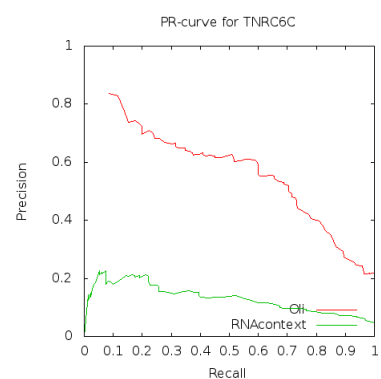

Supplement: Additional file 4 — Precision-recall curves for Oli and RNAcontext on the AURA_dataset. This pdf file shows a PR curve for each RBP, visualizing the performances of Oli and RNAcontext in a 10-fold cross validation. Oli outperforms RNAcontext for most RBPs. [file 1471-2105-15-123-S4.pdf]

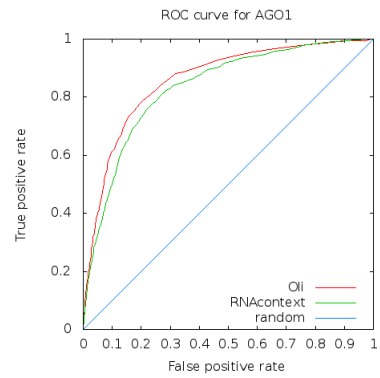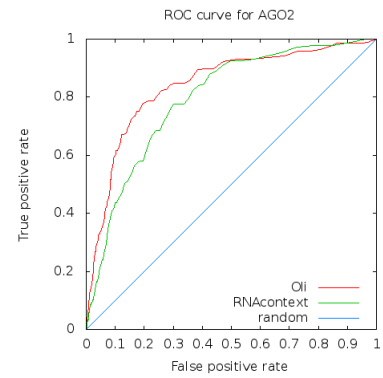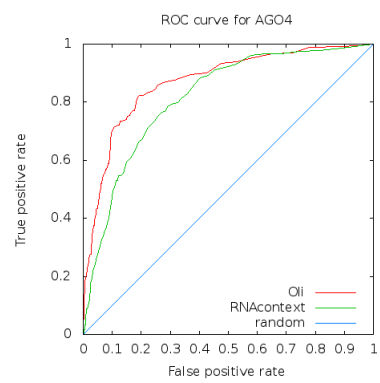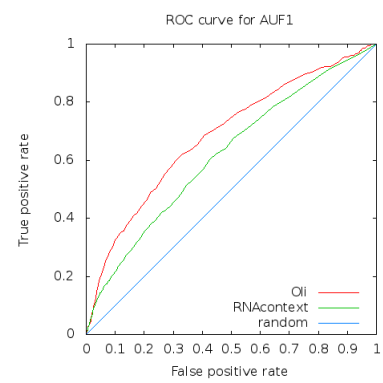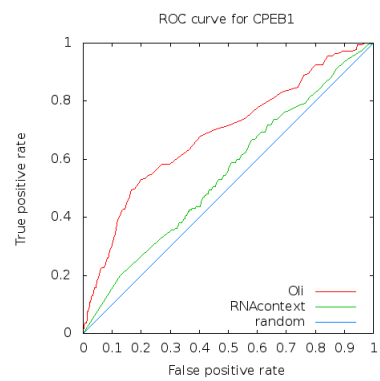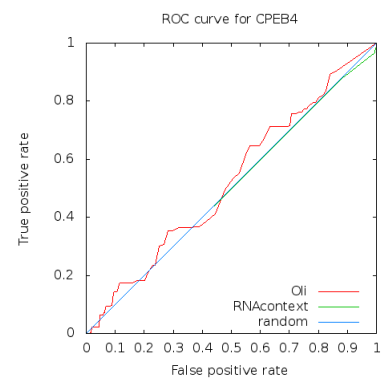

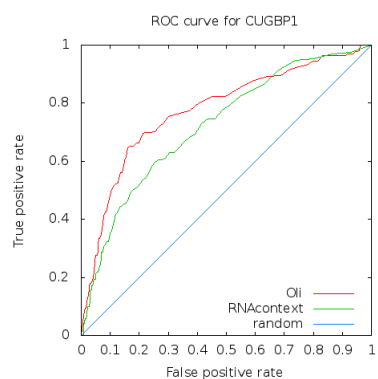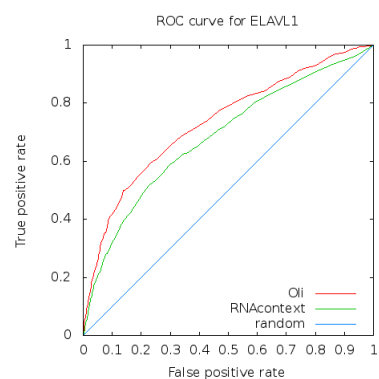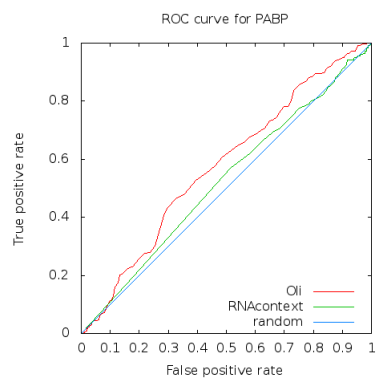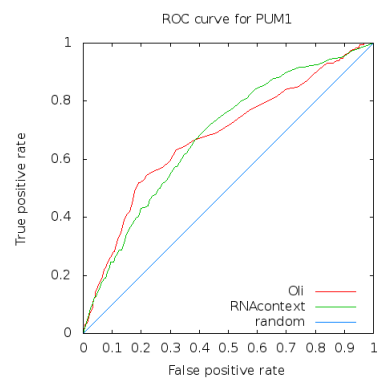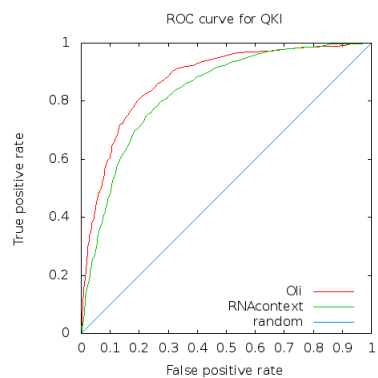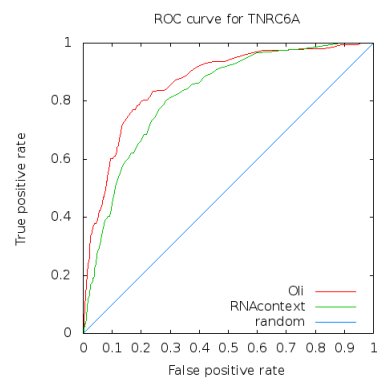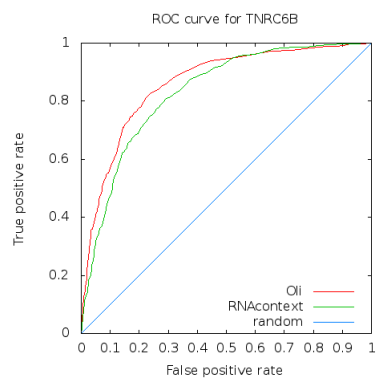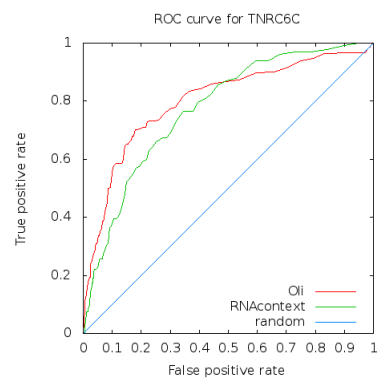

Supplement: Additional file 5 — ROC curves for Oli and RNAcontext on the AURA_dataset. This pdf file shows a ROC curve for each RBP, visualizing the performances of Oli and RNAcontext in a 10-fold cross validation. The curves basically reflect the AUCs in Table 1 and do not show a significant difference in the prediction ability of the two methods. [file 1471-2105-15-123-S5.pdf]
